# The effect of once-weekly insulin icodec vs once-daily basal insulin on physical activity-attributed hypoglycaemia in type 2 diabetes: a post hoc analysis of ONWARDS 1–5

**DOI:** 10.1007/s00125-025-06414-6

**Published:** 2025-04-05

**Authors:** Michael C. Riddell, Simon Heller, Lisbeth Carstensen, Thaís M. Pagliaro Rocha, Sara Kehlet Watt, Vincent C. Woo

**Affiliations:** 1 Muscle Health Research Centre, York University, Toronto, Canada; 2 Department of Oncology and Metabolism, University of Sheffield, Sheffield, UK; 3 Novo Nordisk A/S, Søborg, Denmark; 4 Section of Endocrinology and Metabolism, University of Manitoba, Winnipeg, MB Canada

**Keywords:** Exercise, Hypoglycaemia, Insulin icodec, Once-weekly basal insulin, Physical activity, Type 2 diabetes

## Abstract

**Aims/hypothesis:**

Physical activity increases the risk of hypoglycaemia in individuals with type 2 diabetes when basal or basal-bolus insulin therapy is administered. Once-weekly basal insulins may elevate the risk of physical activity-attributed hypoglycaemia compared with other basal insulins because the administered levels cannot be reduced in anticipation of increased physical activity. This post hoc analysis of five separate randomised trials (ONWARDS 1–5) aimed to examine physical activity-attributed hypoglycaemic episodes in adults with type 2 diabetes receiving either once-weekly basal insulin icodec (herein referred to as ‘icodec’) or once-daily basal insulins.

**Methods:**

The ONWARDS 1–5 Phase 3a randomised controlled trials compared the efficacy and safety of once-weekly basal icodec vs once-daily basal insulin in insulin-naive (ONWARDS 1, 3 and 5) and insulin-experienced (ONWARDS 2 and 4) adults with type 2 diabetes. Participants self-monitored their blood glucose levels using a blood glucose meter and a digital diary. In each trial, suspected hypoglycaemia symptoms triggered additional self-measured blood glucose readings, and values indicative of hypoglycaemia were recorded in the participants’ digital diary. Participants who experienced hypoglycaemic episodes were instructed to note any relation of each episode to physical activity. Hypoglycaemic episodes were classified as alert value (level 1: blood glucose <3.9 but ≥3.0 mmol/l), clinically significant (level 2: blood glucose <3.0 mmol/l) or severe (level 3: cognitive impairment requiring external assistance). The proportions of hypoglycaemic episodes that were attributed to physical activity and the ORs of having a physical activity-attributed hypoglycaemic episode were calculated for the two basal insulin types (once-weekly vs once-daily) for each of the five trials.

**Results:**

Across all trials, there were no consistent differences between icodec and the once-daily insulin comparators in the proportions of hypoglycaemic episodes that were attributed to physical activity; these episodes were mainly alert value or clinically significant hypoglycaemic episodes. In both insulin-naive and insulin-experienced participants, the incidence of physical activity-attributed clinically significant or severe hypoglycaemic episodes was consistently ≤3.0% in ONWARDS 1, 2, 3 and 5. In ONWARDS 4, the incidence of physical activity-attributed hypoglycaemic episodes was numerically higher in both treatment groups (18.6% [icodec] vs 17.9% [insulin glargine U100]), which was expected given the basal-bolus insulin regimen. Across all trials, there were no statistically significant differences in the odds of experiencing a physical activity-attributed clinically significant or severe hypoglycaemic episode with icodec vs once-daily insulin comparators. The frequency of recurrent clinically significant or severe hypoglycaemic episodes in the 24 h after a physical activity-attributed clinically significant or severe hypoglycaemic episode was low, with no such episodes in ONWARDS 1, 3 and 5. In contrast, in ONWARDS 2 and 4, the frequency of recurrent clinically significant hypoglycaemic episodes in the 24 h after a physical activity-attributed clinically significant or severe hypoglycaemic episode was numerically higher with icodec vs the once-daily insulin comparators, whilst no additional severe episodes were reported in any participants across the trials.

**Conclusions/interpretation:**

These findings do not suggest that there is an additional increase in hypoglycaemia risk attributed to physical activity with once-weekly basal icodec vs once-daily basal insulins in adults with type 2 diabetes.

**Trial registration:**

ClinicalTrials.gov NCT04460885 (ONWARDS 1), NCT04770532 (ONWARDS 2), NCT04795531 (ONWARDS 3), NCT04880850 (ONWARDS 4) and NCT04760626 (ONWARDS 5).

**Graphical Abstract:**

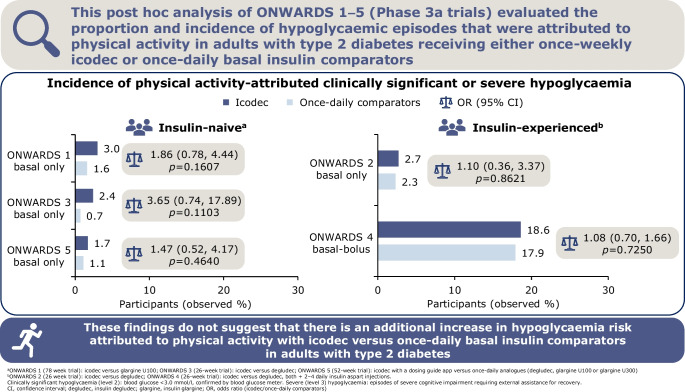

**Supplementary Information:**

The online version contains peer-reviewed but unedited supplementary material available at 10.1007/s00125-025-06414-6.


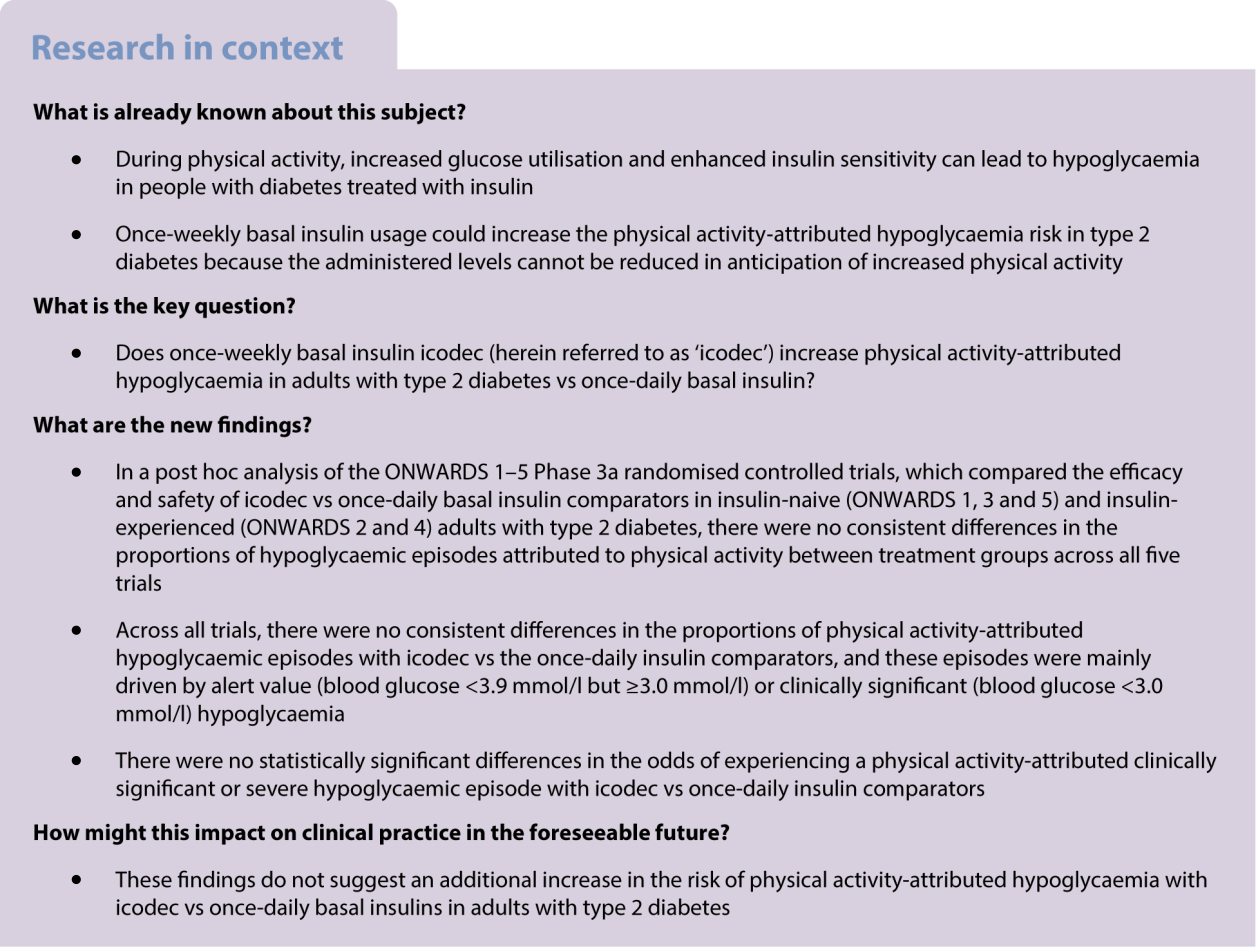


## Introduction

During physical activity, increased glucose requirements and insulin sensitivity can lead to hypoglycaemia in individuals with type 2 diabetes on insulin therapy [1, 2]. Lowering basal insulin on physically active days is challenging since it may have an impact on blood glucose levels throughout the day, but sometimes it is recommended, particularly if the activity is not routine [1, 3]. However, this strategy is not feasible with once-weekly basal insulin.

The efficacy and safety of insulin icodec (herein simply referred to as ‘icodec’), a once-weekly basal insulin analogue, have been evaluated vs once-daily basal insulin comparators in five Phase 3a trials in adults with type 2 diabetes (ONWARDS 1–5 [ClinicalTrials.gov registration nos NCT04460885, NCT04770532, NCT04795531, NCT04880850 and NCT04760626, respectively]) [4–10]. Icodec used without (ONWARDS 1–4) or with (ONWARDS 5) a dosing guide app demonstrated non-inferiority for HbA_1c_ reduction vs once-daily insulin comparators. Subsequent prespecified hierarchical testing demonstrated superior HbA_1c_ reduction for icodec vs once-daily insulin comparators in ONWARDS 1, 2, 3 and 5. Clinically significant or severe hypoglycaemia rates remained below one episode per person-year of exposure in all trials except ONWARDS 4, where the rates were higher, as expected since this trial used a basal-bolus insulin regimen. In ONWARDS 2–5, and in the main 52 week treatment phase (not including the 26 week extension phase) of ONWARDS 1, these rates were numerically higher but not statistically significantly different for icodec vs once-daily insulin comparators [5–9].

This post hoc analysis of ONWARDS 1–5 compared the proportion and incidence of physical activity-attributed hypoglycaemia for once-weekly icodec vs once-daily insulin comparators in adults with type 2 diabetes.

## Methods

The trial designs of ONWARDS 1–5 have been previously published [4–9]. ONWARDS 1–5 were randomised, multicentre trials of insulin-naive (ONWARDS 1, 3 and 5) or insulin-experienced (ONWARDS 2 and 4) men and women (≥18 years of age) from multiple racial and ethnic backgrounds with type 2 diabetes, and no history of recurrent severe hypoglycaemia (ONWARDS 1–4) [5–9]. ONWARDS 5 included real-world elements, such as broad inclusion criteria, choice and dosing of basal insulin comparators at the investigator’s discretion, and individualised trial-site visit schedules. The treatment period lasted either 26 weeks (ONWARDS 2–4) or 52 weeks (ONWARDS 1 and 5). ONWARDS 1 had a 26 week extension phase; the 78 week data are included here. An interactive web response system was used to randomly assign participants 1:1 to once-weekly icodec or a once-daily basal insulin comparator (insulin glargine U100 [ONWARDS 1 and 4], insulin degludec [ONWARDS 2 and 3], or one of insulin degludec, insulin glargine U100 or insulin glargine U300 [ONWARDS 5]). Since ONWARDS 4 was a basal-bolus trial, both icodec and the once-daily basal insulin comparator were administered with 2–4 daily doses of insulin aspart. Full details regarding the randomised treatments and dosing are summarised in electronic supplementary material (ESM) Table 1.

Blood glucose was self-measured by participants using the Accu-Chek blood glucose meter (Roche). Icodec and insulin comparators were titrated weekly to achieve a pre-breakfast self-measured blood glucose (SMBG) target of 4.4–7.2 mmol/l in ONWARDS 1–4. In ONWARDS 5, a dosing guide app was used for icodec, and insulin comparators were titrated per standard practice. Pre-breakfast SMBG was recorded daily in ONWARDS 1–3 and 5, while, in ONWARDS 4, preprandial and bedtime SMBG values were recorded. In all trials, suspected hypoglycaemia symptoms triggered additional SMBG measurements, and values indicative of hypoglycaemia were recorded as hypoglycaemic episodes in the participants’ digital diary (trial-specific software). Participants who reported hypoglycaemic episodes were also asked to indicate any relationship to physical activity.

This post hoc analysis evaluated the proportion of physical activity-attributed hypoglycaemic episodes compared with the total number of hypoglycaemic episodes. Hypoglycaemic episodes were defined as alert value (level 1: blood glucose <3.9 mmol/l but ≥3.0 mmol/l, confirmed by blood glucose meter), clinically significant (level 2: blood glucose <3.0 mmol/l, confirmed by blood glucose meter) and severe (level 3: episodes of severe cognitive impairment requiring external assistance for recovery). Other outcomes analysed were the incidence (percentage of participants experiencing ≥1 hypoglycaemic episode) of physical activity-attributed clinically significant or severe hypoglycaemia, and the proportion of these episodes that were followed by one or more additional clinically significant or severe hypoglycaemic episode(s) in the subsequent 24 h. Hypoglycaemic episodes were reported during the on-treatment period of each trial (ESM Table 2).

The proportions of alert value hypoglycaemic episodes and clinically significant or severe hypoglycaemic episodes were calculated as the number of physical activity-attributed episodes divided by the total number of hypoglycaemic episodes in each trial. For the incidence of physical activity-attributed clinically significant or severe hypoglycaemic episodes, ORs (icodec/once-daily insulin comparator) were estimated using a binary logistic regression model (Logit Link), with treatment, geographical region, sulfonylurea/glinide use (ONWARDS 3) and personal continuous glucose monitoring device use (ONWARDS 2 and 4) as fixed factors. Sex and gender were not considered in the design of this post hoc analysis. In the case of premature trial-treatment discontinuation, hypoglycaemia data for the period that was missing for these participants were imputed using multiple imputation. Two-sided *p* values with no correction for multiplicity were derived to test for no treatment difference, with significance level set to 0.05. For descriptive outputs, R version 4.0.4 was used (https://cran.r-project.org); for statistical analyses, SAS 9.4 (SAS Institute) was used.

## Results

Full population demographics and baseline characteristics, including sex, race and ethnicity, can be found in the primary articles for each of the ONWARDS 1–5 trials [5–9]. Overall, participant baseline characteristics and demographics were numerically similar between icodec and once-daily insulin comparator groups in ONWARDS 1–5 [5–9].

**Fig. 1:**
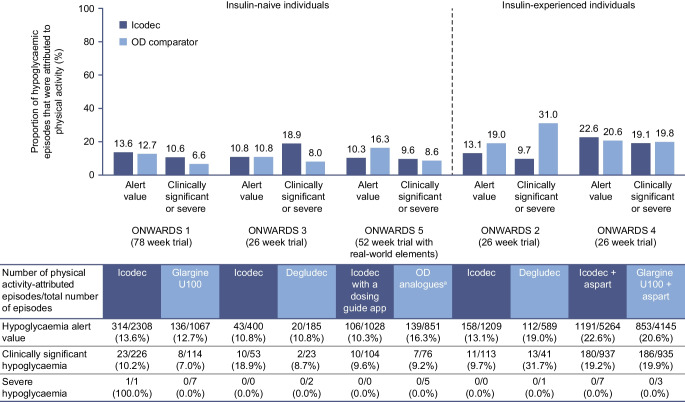
Proportion of hypoglycaemic episodes that were attributed to physical activity during the on-treatment period of ONWARDS 1–5. The on-treatment period was defined as the onset date on or after the first dose of the trial product and no later than the first date of either the follow-up visit, the last date on trial product +5 weeks for once-daily insulin and +6 weeks for once-weekly insulin, or the end date for the in-trial period (defined as the time from randomisation to whichever occurred first of the last direct participant–site contact, withdrawal of informed consent, the last participant–investigator contact before loss to follow-up, or death). Hypoglycaemia alert value was defined as a blood glucose value <3.9 mmol/l but ≥3.0 mmol/l, confirmed by a blood glucose meter; clinically significant hypoglycaemia was defined as a blood glucose value <3.0 mmol/l, confirmed by a blood glucose meter; severe hypoglycaemia was defined as hypoglycaemia with severe cognitive impairment requiring external assistance for recovery. ^a^The choice of the once-daily insulin analogue (insulin degludec, insulin glargine U100 or insulin glargine U300) was made at the discretion of the investigator. Aspart, insulin aspart; degludec, insulin degludec; glargine U100, insulin glargine U100; glargine U300, insulin glargine U300; icodec, insulin icodec; OD, once-daily

In previously insulin-naive participants, 10.3–13.6% of hypoglycaemia alert values and 9.6–18.9% of clinically significant or severe hypoglycaemic episodes were attributed to physical activity in the icodec arms across ONWARDS 1, 3 and 5, while 10.8–16.3% and 6.6–8.6%, respectively, were attributed to physical activity in the once-daily insulin comparator arms of these trials (Fig. 1). In insulin-experienced participants, 13.1–22.6% of hypoglycaemia alert values and 9.7–19.1% of clinically significant or severe hypoglycaemic episodes across ONWARDS 2 and 4 were attributed to physical activity in the icodec arms, while 19.0–20.6% and 19.8–31.0%, respectively, were attributed to physical activity in the once-daily insulin comparator arms (Fig. 1).

Across all trials, there were no consistent differences in the proportions of physical activity-attributed hypoglycaemic episodes with icodec vs the once-daily insulin comparators (Fig. 1). These episodes were mainly alert value or clinically significant hypoglycaemia, with one physical activity-attributed severe hypoglycaemic episode reported in the icodec arm of ONWARDS 1 (Fig. 1), which had other possible contributing factors reported, such as diet change.

**Fig. 2:**
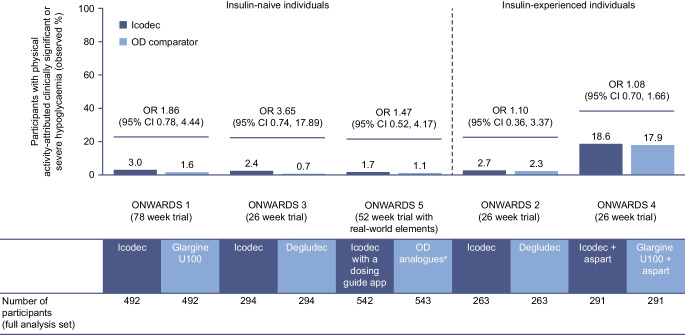
Observed incidence of physical activity-attributed clinically significant or severe hypoglycaemia with icodec vs once-daily insulin comparators during the on-treatment period of ONWARDS 1–5. The table presents the number of participants in each treatment arm that were included in the analyses that are presented in the bar graph. The on-treatment period was defined as the onset date on or after the first dose of trial product and no later than the first date of either the follow-up visit, the last date on trial product +5 weeks for once-daily insulin and +6 weeks for once-weekly insulin, or the end date for the in-trial period (defined as the time from randomisation to whichever occurred first of the last direct participant–site contact, withdrawal of informed consent, the last participant–investigator contact before loss to follow-up, or death). The incidence of hypoglycaemia was analysed using a binary logistic regression model, with treatment, geographical region, sulfonylurea/glinide use (ONWARDS 3) and personal continuous glucose monitoring device use (ONWARDS 2 and 4) as fixed factors. Missing data were imputed using multiple imputations. Clinically significant hypoglycaemia was defined as a blood glucose value <3.0 mmol/l, confirmed by a blood glucose meter; severe hypoglycaemia was defined as hypoglycaemia with severe cognitive impairment requiring external assistance for recovery. ORs (icodec/once-daily insulin comparator) are shown; there were no statistically significant differences between treatment arms in each trial (two-sided *p* values for the test of no treatment difference [with no correction for multiplicity] were *p*=0.1607, *p*=0.1103 and *p*=0.4640 for ONWARDS 1, ONWARDS 3 and ONWARDS 5, respectively, and *p*=0.8621 and *p*=0.7250 for ONWARDS 2 and ONWARDS 4, respectively). ^a^The choice of the once-daily insulin analogue (degludec, glargine U100 or glargine U300) was made at the discretion of the investigator. Aspart, insulin aspart; degludec, insulin degludec; glargine U100, insulin glargine U100; glargine U300, insulin glargine U300; icodec, insulin icodec; OD, once-daily

In both insulin-naive and insulin-experienced participants, the incidence of physical activity-attributed clinically significant or severe hypoglycaemic episodes was consistently ≤3.0% in ONWARDS 1–3 and 5. In ONWARDS 4, the incidence was numerically higher in both treatment groups (icodec vs insulin glargine U100: 18.6% vs 17.9%), which was expected given the basal-bolus insulin regimen used in this trial (Fig. 2).

Across all trials, there were no statistically significant differences in the odds of experiencing a physical activity-attributed clinically significant or severe hypoglycaemic episode with icodec vs once-daily insulin comparators (Fig. 2).

The frequency of recurrent clinically significant or severe hypoglycaemic episodes in the 24 h after a physical activity-attributed clinically significant or severe hypoglycaemic episode was low, with no such episodes being reported in ONWARDS 1, 3 and 5. In ONWARDS 2 and 4, this frequency was numerically higher with icodec vs the once-daily insulin comparator; no additional severe episodes were reported in any participants across the trials (ESM Table 3).

## Discussion

In this post hoc analysis of ONWARDS 1–5, most physical activity-attributed hypoglycaemic episodes were categorised as alert value or clinically significant, with no discernible trends across treatment arms. The incidence of physical activity-attributed clinically significant or severe hypoglycaemia was generally low in ONWARDS 1–5 and was not statistically significantly different between icodec and once-daily insulin comparators. A recent meta-analysis suggested that once-weekly icodec may increase the rate of clinically significant hypoglycaemia compared with once-daily basal insulins, particularly if not carefully titrated [11]. Therefore, it could be hypothesised that physical activity-attributed hypoglycaemic episodes may also be higher with icodec vs once-daily insulin comparators. However, the increased rates of clinically significant hypoglycaemia with icodec that were observed in the meta-analysis do not appear to be attributed to physical activity, as shown in the current study. Furthermore, the frequency of recurrent hypoglycaemia in the 24 h after a physical activity-attributed clinically significant or severe hypoglycaemic episode was also low, with no additional severe hypoglycaemic episodes reported in the icodec or insulin comparator arms.

The association between physical activity and hypoglycaemia risk in type 1 diabetes is well recognised [12, 13], yet studies about this phenomenon in type 2 diabetes are limited. In 344 insulin-experienced veterans with stable type 2 diabetes, ~10% of all participant-documented hypoglycaemic episodes were attributed to exercise [14]. Our results build on other findings that hypoglycaemia risk with exercise is relatively low for individuals with type 2 diabetes on basal insulin [15] and it is reassuring that there were no consistent differences in the physical activity-attributed hypoglycaemia risk with a once-weekly basal insulin compared with daily basal insulin.

Strengths of this study are the large number of participants enrolled in each trial and the long trial durations. However, we also recognise our research limitations. There was no specific definition for ‘physical activity’ in ONWARDS 1–5, possibly leading to inconsistent attributions of hypoglycaemic episodes to physical activity. Additionally, data on the participants’ physical activity levels before or during the trial were not collected, nor were details on how participants managed or mitigated physical activity-attributed hypoglycaemia. This precluded the estimation of rates for physical activity-attributed hypoglycaemic episodes as the duration and intensity of physical activity were unknown. The relatively low incidence of hypoglycaemic episodes, especially among insulin-naive participants, limited the robustness of our analysis. Another limitation was the open-label trial designs of ONWARDS 1, 2, 4 and 5, meaning that participants’ biases may have affected the self-reported data. Furthermore, as the potential effects of sex or gender on the outcomes were not specifically accounted for in this post hoc analysis, these variables may have confounded the results. An ongoing Phase 1 trial of icodec (ClinicalTrials.gov registration no. NCT06288412) will partially address these limitations by providing additional safety data for icodec during and after exercise and prolonged fasting in insulin-experienced individuals with type 2 diabetes.

Overall, these findings do not suggest that there is an additional increase in the hypoglycaemia risk attributed to physical activity with once-weekly icodec vs once-daily basal insulins in adults with type 2 diabetes.

## Supplementary Information

Below is the link to the electronic supplementary material.

Supplementary file1 (PDF 478 KB)

## Data Availability

Individual participant data will be shared in datasets in a de-identified or anonymised format. Shared data will include datasets from clinical research sponsored by Novo Nordisk and completed after 2001 for product indications approved in the EU and the USA. The study protocol and redacted clinical study report will be made available according to Novo Nordisk’s data-sharing commitments. These data will be available permanently after research completion and after approval of product and product use in both the EU and the USA (no end date). Data will be shared with bona fide researchers submitting a research proposal requesting access to data for use as approved by the Independent Review Board (IRB) according to the IRB charter (see www.novonordisk-trials.com). These data can be accessed via an access request proposal form; the access criteria can be found at www.novonordisk-trials.com. The data will be made available on a specialised SAS data platform.

## Abbreviation

SMBG: Self-measured blood glucose

## Authors’ relationships and activities

MCR reports consulting fees from Eli Lilly, the Jaeb Center for Health Research, Zealand Pharma and Zucara Therapeutics; speaker fees from Dexcom Canada, Eli Lilly, Novo Nordisk and Sanofi Diabetes; and stock options from Supersapiens and Zucara Therapeutics. SH reports consultancy fees from Zealand Pharma and Zucara Therapeutics; fees for speaker panel involvement with Novo Nordisk; data monitoring and safety board participation with Eli Lilly; and research support from Dexcom. LC, TMPR and SKW are employees and shareholders of Novo Nordisk. VCW reports speaker fees and advisory board meeting involvement for Boehringer Ingelheim, Eli Lilly and Novo Nordisk.
