# Supplementary material for: The effect of once-weekly insulin icodec vs once-daily basal insulin on physical activity-attributed hypoglycaemia in type 2 diabetes: a post hoc analysis of ONWARDS 1–5
Source: Diabetologia. 2025 Apr 5;68(7):1416–22. doi: 10.1007/s00125-025-06414-6 (PMC12176998; doi:10.1007/s00125-025-06414-6)
Supplement: Supplementary file 1 — Supplementary file1 (PDF 478 KB) [file 125_2025_6414_MOESM1_ESM.pdf]

Electronic supplementary material

The effect of once-weekly insulin icodec versus once-daily basal insulin on physical activity-attributed hypoglycaemia in type 2 diabetes: a post hoc analysis of ONWARDS 1–5

Michael C. Riddell • Simon Heller • Lisbeth Carstensen • Thaís M. Pagliaro Rocha • Sara Kehlet Watt • Vincent C. Woo

ESM Table 1 Overview of ONWARDS 1–5 trial designs [1-6]

	Insulin-naive adults with T2D			Insulin-experienced adults with T2D	
	**ONWARDS 1**	**ONWARDS 3**	**ONWARDS 5**	**ONWARDS 2**	**ONWARDS 4**
Intervention arm (number of randomised participants)	OW icodec (492)	OW icodec + OD placebo (294)	OW icodec with a dosing guide app (542)	OW icodec (263)	OW icodec + aspart 2–4 times daily (291)
Comparator arm (number of randomised participants)	OD glargine U100 (492)	OD degludec + OW placebo (294)	OD basal insulin analogues: degludec (378), glargine U100 (96), glargine U300 (69)[a]	OD degludec (263)	OD glargine U100 + aspart 2–4 times daily (291)
Non-insulin glucose-lowering therapies prior to trial	Stable doses of glucose-lowering therapies[b] ≥90 days before screening were permitted				
Non-insulin glucose-lowering therapies during trial	Participants could continue pre-trial glucose-lowering therapies (except for sulfonylureas and glinides)	Participants could continue pre-trial glucose-lowering therapies[c]		Participants could continue pre-trial glucose-lowering therapies (except for sulfonylureas and glinides)	

Insulin initiation dosage	Icodec: 70 U/week<br>Glargine U100:<br>10 U/day	Icodec: 70 U/week<br>Degludec: 10 U/day	Recommended icodec starting dosage: 70 U/week<br>OD basal insulin analogues initiated in accordance with the local approved label	Icodec: one-time additional 50% dose of icodec administered for the first injection only. From week 2, participants received the calculated OW icodec dose<br>Degludec dose determined by the local approved label	Icodec: one-time additional 50% dose of icodec administered for the first injection only. From week 2, participants received the calculated OW icodec dose<br>Glargine U100 determined by the local approved label<br>Participants were switched from their previous bolus insulin to aspart on a unit-to-unit basis per meal

Icodec, degludec and aspart were manufactured by Novo Nordisk A/S; glargine U100 and U300 were manufactured by Sanofi. [a]The choice of the OD basal insulin analogue was made at the discretion of the investigator. [b]Included metformin, sulfonylureas, meglitinides (glinides), DPP-4 inhibitors, SGLT2is, thiazolidinediones, alpha-glucosidase inhibitors, oral combination products for the allowed individual glucose-lowering agents, and oral or injectable GLP-1 RAs. [c]Doses of sulfonylureas and glinides were administered at a reduced dose (50%) at randomisation at the discretion of the investigator. Aspart, insulin aspart; degludec, insulin degludec; DPP-4, dipeptidyl peptidase 4; glargine U100, insulin glargine U100; glargine U300, insulin glargine U300; GLP-1 RA, glucagon-like peptide-1 receptor agonist; icodec, insulin icodec; OD, once-daily; OW, once-weekly; SGLT2i, sodium-glucose co-transporter-2 inhibitor; T2D, type 2 diabetes; U, units

ESM Table 2 Definitions for the ‘on-treatment period’ and the ‘in-trial period’

On-treatment period	In-trial period
Defined as the onset date on or after the first dose of trial product and no later than the first date of either the follow-up visit, the last date on trial product +5 weeks for once-daily basal insulin and +6 weeks for once-weekly icodec, or the end-date for the in-trial period	Defined as the time from randomisation to whichever occurs first of: last direct participant–site contact, withdrawal of informed consent, the last participant–investigator contact before loss to follow-up, or death

ESM Table 3 Proportion of clinically significant or severe hypoglycaemic episodes attributed to physical activity with at least one additional clinically significant or severe hypoglycaemic episode in the following 24 hours

Number of physical activity-attributed clinically significant or severe hypoglycaemic episodes with recurrent episodes/total number of physical activity-attributed clinically significant or severe hypoglycaemic episodes	Insulin icodec		Once-daily comparator	
	Clinically significant hypoglycaemia	Severe hypoglycaemia	Clinically significant hypoglycaemia	Severe hypoglycaemia
ONWARDS 1 (basal initiation; 78 week trial)	0/23 (0.0%)	0/1 (0.0%)	0/8 (0.0%)	0/0 (0.0%)
ONWARDS 3 (basal initiation; 26 week trial)	0/10 (0.0%)	0/0 (0.0%)	0/2 (0.0%)	0/0 (0.0%)
ONWARDS 5 (basal initiation; 52 week trial with real-world elements)	0/10 (0.0%)	0/0 (0.0%)	0/7 (0.0%)	0/0 (0.0%)
ONWARDS 2 (basal switch; 26 week trial)	3/11 (27.3%)	0/0 (0.0%)	0/13 (0.0%)	0/0 (0.0%)
ONWARDS 4 (basal-bolus; 26 week trial)	27/180 (15.0%)	0/0 (0.0%)	16/186 (8.6%)	0/0 (0.0%)

Clinically significant hypoglycaemia was defined as a blood glucose value <3.0 mmol/L, confirmed by a blood glucose meter; severe hypoglycaemia was defined as hypoglycaemia with severe cognitive impairment requiring external assistance for recovery.

References

1. Philis-Tsimikas A, Bajaj HS, Begtrup K, et al. (2023) Rationale and design of the phase 3a development programme (ONWARDS 1-6 trials) investigating once-weekly insulin icodec in diabetes. Diabetes Obes Metab 25(2):331–341. https://doi.org/10.1111/dom.14871
2. Rosenstock J, Bain SC, Gowda A, et al. (2023) Weekly Icodec versus Daily Glargine U100 in Type 2 Diabetes without Previous Insulin. N Engl J Med 389(4):297–308. https://doi.org/10.1056/NEJMoa2303208
3. Philis-Tsimikas A, Asong M, Franek E, et al. (2023) Switching to once-weekly insulin icodec versus once-daily insulin degludec in individuals with basal insulin-treated type 2 diabetes (ONWARDS 2): a phase 3a, randomised, open label, multicentre, treat-to-target trial. Lancet Diabetes Endocrinol 11(6):414–425. https://doi.org/10.1016/s2213-8587(23)00093-1
4. Lingvay I, Asong M, Desouza C, et al. (2023) Once-weekly insulin icodec vs once-daily insulin degludec in adults with insulin-naive type 2 diabetes: the ONWARDS 3 randomized clinical trial. JAMA 330(3):228–237. https://doi.org/10.1001/jama.2023.11313
5. Mathieu C, Ásbjörnsdóttir B, Bajaj HS, et al. (2023) Switching to once-weekly insulin icodec versus once-daily insulin glargine U100 in individuals with basal-bolus insulin-treated type 2 diabetes (ONWARDS 4): a phase 3a, randomised, open-label, multicentre, treat-to-target, non-inferiority trial. Lancet 401(10392):1929–1940. https://doi.org/10.1016/s0140-6736(23)00520-2
6. Bajaj HS, Aberle J, Davies M, et al. (2023) Once-weekly insulin icodec with dosing guide app versus once-daily basal insulin analogues in insulin-naive type 2 diabetes (ONWARDS 5) : a randomized trial. Ann Intern Med 176(11):1476–1485. https://doi.org/10.7326/m23-1288
